# Rapid diagnosis and comprehensive bacteria profiling of sepsis based on cell-free DNA

**DOI:** 10.1186/s12967-019-02186-x

**Published:** 2020-01-06

**Authors:** Pei Chen, Shuo Li, Wenyuan Li, Jie Ren, Fengzhu Sun, Rui Liu, Xianghong Jasmine Zhou

**Affiliations:** 1 School of Mathematics, South China University of Technology, Guangzhou, 510640 China; 2 Department of Pathology and Laboratory Medicine, David Geffen School of Medicine, University of California at Los Angeles, Los Angeles, 90095 USA; 3 Quantitative and Computational Biology Program, Department of Biological Sciences, University of Southern California, Los Angeles, CA 90089 USA; 4 Google Research, Mountain View, CA USA

**Keywords:** Sepsis, Bacteremia, Rapid diagnosis, Cell-free DNA sequence, Bacterial co-occurrence network, Bacteria profiling

## Abstract

**Background:**

Sepsis remains a major challenge in intensive care units, causing unacceptably high mortality rates due to the lack of rapid diagnostic tools with sufficient sensitivity. Therefore, there is an urgent need to replace time-consuming blood cultures with a new method. Ideally, such a method also provides comprehensive profiling of pathogenic bacteria to facilitate the treatment decision.

**Methods:**

We developed a Random Forest with balanced subsampling to screen for pathogenic bacteria and diagnose sepsis based on cell-free DNA (cfDNA) sequencing data in a small blood sample. In addition, we constructed a bacterial co-occurrence network, based on a set of normal and sepsis samples, to infer unobserved bacteria.

**Results:**

Based solely on cfDNA sequencing information from three independent datasets of sepsis, we distinguish sepsis from healthy samples with a satisfactory performance. This strategy also provides comprehensive bacteria profiling, permitting doctors to choose the best treatment strategy for a sepsis case.

**Conclusions:**

The combination of sepsis identification and bacteria-inferring strategies is a success for noninvasive cfDNA-based diagnosis, which has the potential to greatly enhance efficiency in disease detection and provide a comprehensive understanding of pathogens. For comparison, where a culture-based analysis of pathogens takes up to 5 days and is effective for only a third to a half of patients, cfDNA sequencing can be completed in just 1 day and our method can identify the majority of pathogens in all patients.

## Background

Sepsis, a life-threatening emergency condition arising from various infections of skin, lung, abdomen, and urinary tract, is a challenge for hospitals and causes unacceptably high mortality rates in intensive care medicine [1, 2]. In recent decades, great efforts have been devoted to sepsis research, and novel therapies have been developed against pathogenic bacteria. To guarantee an effective treatment strategy, it is vital to quickly and accurately detect the bacteria or other pathogens that cause the sepsis. According to a recent guideline, deploying an appropriate antibiotic therapy as early as possible (preferably within 1 h) is crucial for septic patients [3]. For example, in septic shock patients with hypotension, the risk of mortality increases by 7.6% with every hour of delay in administering effective antibiotic therapy [4]. However, the standard procedure of pathogen detection for sepsis patients is culture-based (e.g., making blood cultures after a confirmatory test). Since this method relies on bacterial growth, a significant period of time is required (up to 5 days) [3, 5]. Moreover, it sometimes fails to identify the specific pathogens for a sepsis patient. Only a third to a half of people with sepsis yield positive results in blood cultures [6]. Therefore, a more rapid approach to diagnosing sepsis samples and comprehensive bacteria profiling is urgently required.

Cell-free DNA (cfDNA) refers to small fragments of freely circulating DNA detectable in almost all body fluids, including plasma and serum. Most of these DNA fragments are human, having been shed into the bloodstream during the processes of cell apoptosis [7] and cell necrosis [8]. However, cfDNA also includes fragments from other life forms such as bacteria, viruses, fungi [9–11], and even plants via food consumption [12]. With the development of next-generation sequencing (NGS) technology, cfDNA is a promising, noninvasive tool for the early detection of several human diseases. It has been used to find predictive biomarkers for cancer [8, 13–15], as a diagnostic tool for injury [16] and as a way of monitoring organ transplant rejection in real time [10]. Recently, high levels of cfDNA in blood are being observed as a side effect of more and more infectious diseases [17, 18]. These and other uses of cfDNA in plasma represent a rapidly developing field in biomedicine.

**Fig. 1:**
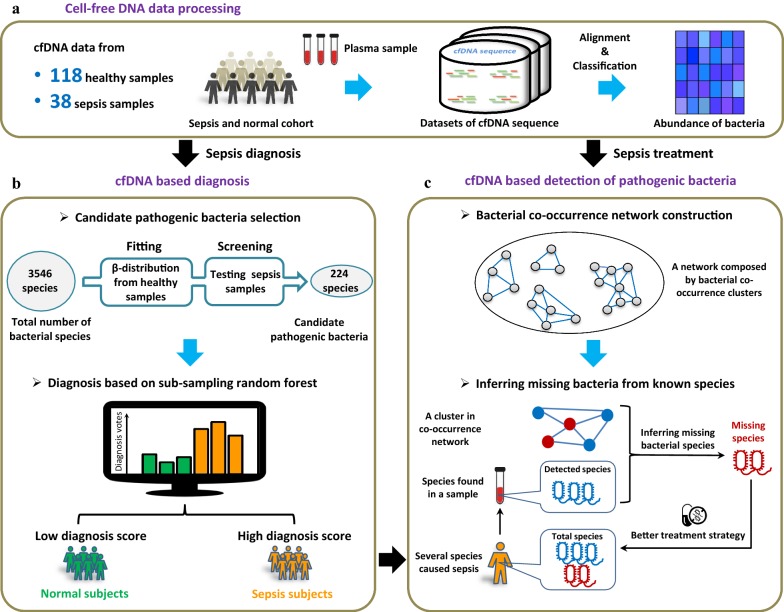
An illustration of our approach to sepsis diagnosis and bacteria inference based on cell-free DNA (cfDNA). **a** We used two public cfDNA datasets to obtain 38 sepsis and 118 healthy samples. All human reads were removed from the datasets using Bowtie2. Through alignment and classification, the normalized abundances of bacteria were estimated from the remaining non-human reads using Centrifuge [27]. **b** Our diagnosis strategy is a two-step procedure based solely on cfDNA from blood. First, we selected candidate pathogenic bacterial species through statistical analysis (see “Methods”). Second, a Random Forest is used to calculate a diagnosis score for each sample. **c** Due to the limited volume of a blood sample, not all bacterial species will be identified in cfDNA sequencing data. Using the bacterial co-occurrence network, we developed a method to infer unobserved bacterial species

In this study, we achieved two aims: (1) we developed a cfDNA-based strategy that can rapidly diagnose sepsis patients and accurately profile the bacteria responsible; and (2) we constructed a sepsis-specific bacterial co-occurrence network to infer unobserved bacterial species from the cfDNA sequencing data. Towards the first aim, cfDNA was isolated and sequenced from the blood samples (Fig. 1a) of healthy and sepsis cohorts. Based on these data, candidate pathogenic bacteria were identified and ranked by statistical models. Our rapid sepsis diagnosis method achieved an area under the ROC curve (AUC) of 93%. Our second aim of identifying missing bacteria is of practical importance, because not all infection-causing bacteria may be detected in cfDNA due to the limited volume of a blood sample. An incomplete bacteria profile may bias the treatment decision. We validated our method for inferring missing bacteria through simulation experiments, and found the approach to be both effective and robust. In particular, when some bacteria species were randomly removed from a simulated sample, our method could recall those species at a high rate. In fact, even when 80% of species in the sample are randomly removed, the recovery rate among all bacterial species present is still 60%. This method may therefore provide a comprehensive understanding of sepsis-causing and infection-related bacterial species, greatly facilitating therapeutic decisions for sepsis treatment.

## Materials and methods

### Data collection and processing

The cfDNA sequencing data used in this study were taken from 38 sepsis and 118 healthy samples. The raw sequencing reads were derived from three previously published data sources: 38 sepsis and 15 healthy samples from the European Nucleotide Archive (ENA, study 1, No. PRJEB13247 [19]), 103 healthy samples from the European Genome-phenome Archive (EGA, study 2, No. EGAS00001001754 [20]), 165 asymptomatic samples and 187 symptomatic from the European Nucleotide Archive (ENA, study 3, No. PRJNA507824) [21]. Samples from above studies were taken from plasma, then whole genome and single-end were sequenced. The raw reads from ENA(PRJEB13247) and ENA(PRJNA507824) were cleaned of human-like reads and reads with low complexity stretches. For the EGA data, the raw sequencing reads were preprocessed to remove human and human-like reads using the fast alignment program Bowtie2 [22].

### Read alignment and quantification

The nonhuman sequencing reads were aligned to a microbial genome sequence database using Centrifuge [23], an open-source microbial classification engine that enables rapid and accurate labeling of reads and quantification of species. Specifically, the mapping was based on a database of compressed microbial sequences provided by Centrifuge (https://ccb.jhu.edu/software/centrifuge/manual.shtml).

Traversing up a taxonomic tree, Centrifuge maps reads to taxon nodes and assigns a “species abundance” to each taxonomic category. The abundances are the estimated fractions $\alpha = \left( {\alpha_{1} ,\alpha_{2} , \ldots ,\alpha_{S} } \right)$ that maximize a likelihood function; i.e.,

1 $$\alpha = \arg_{\alpha } Max\left( L \right)$$

with the likelihood $L$ given by

2 $$L\left( \alpha \right) = \mathop \prod \limits_{i = 1}^{R} \mathop \sum \limits_{j = 1}^{S} \left( {\frac{{\alpha_{j} l_{j} }}{{\mathop \sum \nolimits_{k}^{s} \alpha_{k} l_{k} }}C_{ij} } \right)$$

*R* is the number of the reads, *S* is the number of species, $\alpha_{j}$ is the abundance of species *j* ($\mathop \sum \nolimits_{j = 1}^{S} \alpha_{j} = 1, 0 < \alpha_{j} < 1$), and $l_{j}$ is the average length of the genomes of species *j*. The coefficient $C_{ij}$ is 1 if read *i* is classified to species *j*, and 0 otherwise. The abundance vector α is obtained through an expectation maximization (EM) procedure.

Through this procedure, two bacterial abundance matrices were obtained from the sepsis and healthy samples. For each matrix, a row represents a bacterial species, and a column represents a sample.

### Identification of candidate pathogenic bacteria

In order to detect an abnormal bacterial abundance in a cfDNA sample, we need to first establish the background distribution of abundances under healthy conditions. We fit the expected abundance of each species in healthy samples with a Beta distribution. (This is a family of continuous probability distributions defined on the interval [0, 1] and parametrized by two positive parameters.) Specifically, for each bacterial species *j*, its observed abundance values across a training set of healthy samples were used to fit a species-specific Beta distribution defined by the parameters *a*_*j*_ and *b*_*j*_.

To determine if bacterial species *j* is a candidate pathogen, we compare the abundance value *α*_*j*_ from a new sample (healthy or sepsis) to the Beta distribution. Specifically, we calculate the probability *P* to observe an abundance higher than *α*_*j*_ assuming that the sample is healthy:

3 $$P\left( {x \ge \alpha_{j} |a,b} \right) = \frac{{\mathop \smallint \nolimits_{{\alpha_{j} }}^{1} u^{{a_{j} - 1}} \left( {1 - u} \right)^{{b_{j} - 1}} {\text{d}}u}}{{\mathop \smallint \nolimits_{0}^{1} u^{{a_{j} - 1}} \left( {1 - u} \right)^{{b_{j} - 1}} {\text{d}}u}} ,$$

If *P* is very small, then we can reject the hypothesis that the observed abundance of this bacterial species in this sample was produced by the Beta distribution determined under healthy conditions, and hence conclude that the abundance of this species is abnormally high and a candidate pathogen for sepsis. A bacterial species is classified as a candidate pathogen in our study if it meets this condition for at least one of the sepsis samples.

### Random Forest with balanced subsampling

Random Forest is an effective classification method that generates many binary decision trees [24] and aggregates their results. Each decision tree is trained on a bootstrapped subsample of the original training data, and searches for decision thresholds that effectively split the sample into classes among a randomly selected subset of the input features (in our case, all bacterial species that are pathogen candidates). The final decision of the Random Forest is reached by aggregating the decisions of each tree with majority vote. Random Forest and its extension are widely used in the recent research of disease diagnosis. Ada, a variates of Random Forest was used in cfDNA discrimination of cancer types [25]. A sparse regression–based random forest was designed to predict the Alzheimer’s disease [26].

Due to the imbalanced sizes of the healthy and sepsis samples, a traditional Random Forest may yield biased predictions. Therefore, we employ repeated balanced sub-sampling to build our sepsis diagnosis model. This technique divides the training data into multiple randomized sub-samples, while ensuring that the classes in each sub-sample are equal in size. In our case, we generated subsamples of size 30, where 15 are from healthy patients and 15 are from sepsis patients. For a sub-sampling group of training sets, a decision tree was fitted. We constructed a forest of 500 binary decision trees with balanced subsampling strategy, in this way generating an unbiased diagnosis model from the aggregative decision.

### Co-occurrence network inference

The bacterial DNA fragments in human blood may be shed from many species [27]. These bacteria are naturally present throughout the human body, from skin to viscera, and even in environments previously considered sterile such as blood in circulation [28]. It is of great importance to know how DNA fragments from different species with different habitats come together. Strong inter-taxa associations in the data may indicate a community (even including different domains of life, such as Bacteria and Archaea) originating in a common niche space, or perhaps direct symbioses between community members. Such information is particularly valuable in environments where the basic ecology and life history strategies of many microbial taxa remain unknown. Besides, exploring co-occurrence patterns between different microorganisms can help identify potential biotic interactions, habitat affinities, or shared physiologies that could guide more focused studies or experimental settings [29]. In particular, can we infer the existence of one bacterial species from the occurrence of other species in a blood sample?

A co-occurrence network is a visualization of relationships among entities that usually appear together. For example, it can be used to study the distribution of biotic populations [30], to predict cancer risk [31] or to analyze text collections [32]. We constructed a cfDNA-based bacteria co-occurrence network, where two species are considered co-occurring if their abundances estimated from cfDNA are strongly correlated. Each node in the network represents a bacterial species, while each edge stands for a co-occurring relationship.

In order to construct a bacterial co-occurrence network, we first generated two matrices: (1) the observed abundance matrix *O* (with *n* species, *m* samples); and (2) the expected abundance matrix *N* (also with *n* species, *m* samples). The latter is filled within each local sample as predicted by a regional species distribution model, which is called a leave-one-out LOESS model [29]. An $n \times n$ covariance matrix Σ is calculated from either *O* or *N* by comparing rows (i.e., the abundances of 2 species across all samples). From the inverse of this covariance matrix, the partial correlation $C_{ij}$ between a pair of bacterial species is calculated as follows:

4 $$C_{ij} \left( M \right) = \frac{{ - \mathop \sum \nolimits_{ij}^{ - 1} \left( M \right)}}{{\sqrt {\mathop \sum \nolimits_{ii}^{ - 1} \left( M \right)\mathop \sum \nolimits_{jj}^{ - 1} \left( M \right)} }}$$

where *M* is an $n \times m$ input matrix (*O* or *N*).

Both *C*(*O*) and *C*(*N*) were computed based on Eq. (4). Then the standard effect of correlation between *O* and *N* was calculated by rescaling *C*(*O*)*, C*(*N*). Finally, significant associations were found by calculating the *p* value of the correlation coefficient for each pair of species *i* and *j*, with the null hypothesis that the observations are uncorrelated. Finally, our co-occurrence network was generated by placing edges between each pair of bacterial species with a significant link. The detailed algorithm of network construction is described in [33].

## Results

### A novel strategy for rapid sepsis diagnosis based on cfDNA

**Fig. 2:**
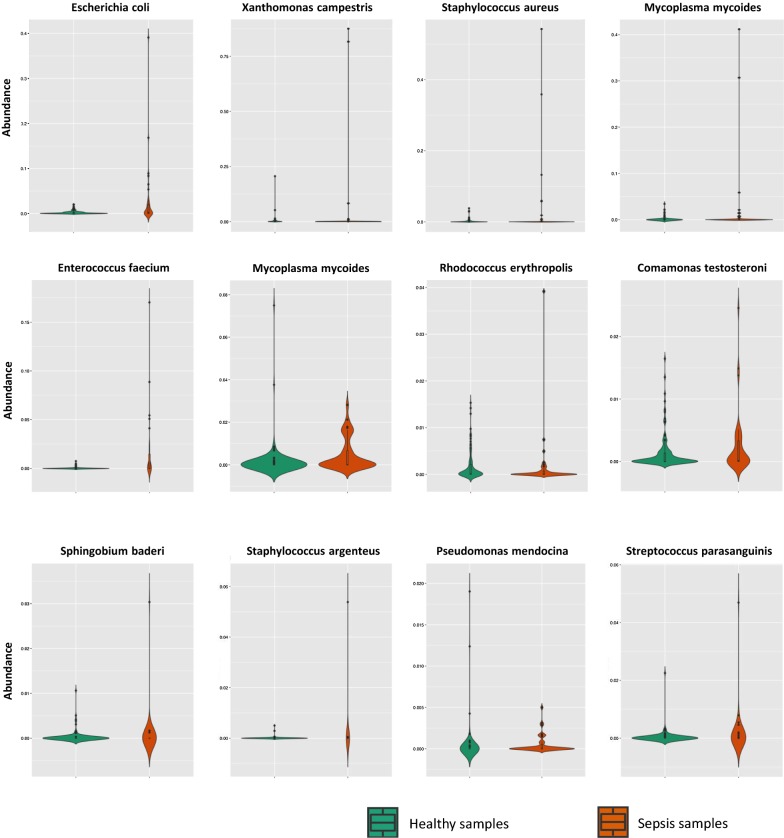
Differential abundances of some candidate pathogenic bacterial species in heathy and sepsis samples. The distributions of bacterial abundances for 12 candidate pathogens are visualized as violin plots

Following the procedures shown in Fig. 1a, b, we developed a two-step approach for rapid sepsis diagnosis, which has been validated by the cross validation and an independent dataset. For the cross-validation, first, we identified 3546 bacterial species through alignment and classification of cfDNA sequencing reads from 118 healthy and 38 sepsis samples. A list of corresponding *P*-values by *T*-test, which were generated for measuring the difference between sepsis and healthy samples from study 1 (No. PRJEB13247) and study 2 (No. EGAS00001001754) respectively, was provided as Additional file 1: Table S1. All samples are randomly partitioned into two groups: 2/3 (78 healthy samples and 25 sepsis samples) for training and 1/3 (40 healthy samples and 13 sepsis samples) for testing. For each species, we fit a Beta distribution based on its bacterial-abundance vector with 78 elements from the healthy training samples. Then the 25 abundances from the sepsis training samples were tested one by one against the Beta distribution, to generate 25 *P*-values. Here a species was considered as a candidate pathogen if at least one satisfying *P*-value < 0.01. By such a filtering procedure, about 220 candidate pathogenic bacteria were selected. Figure 2 shows some examples of these candidate pathogens, which have significantly different distributions between the bacterial abundances of healthy and sepsis samples.

**Fig. 3:**
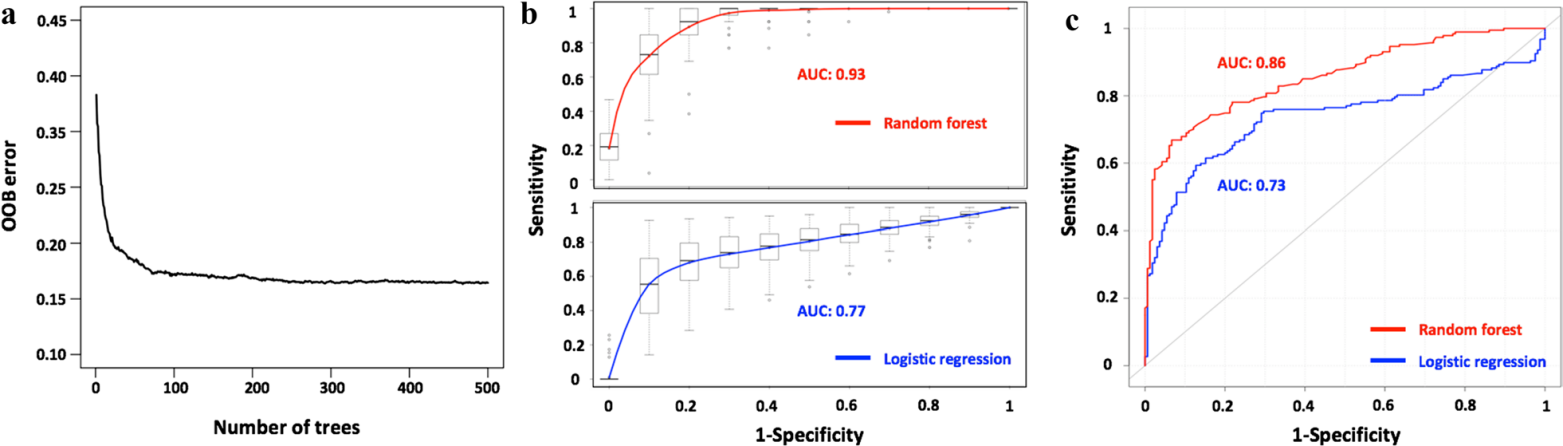
The performance of a Random Forest classifier with balanced subsampling for identifying sepsis samples and healthy samples. **a** The out-of-bag error converges to 0.16, if the number of decision trees is over 100. **b** The average AUC curves for our diagnosis strategy (red) and a logistic regression scheme (blue) based on the one-third of the samples reserved for testing the model. **c** The AUC curves of our diagnosis strategy (red) and a logistic regression scheme (blue) based on an independent dataset for validating the proposed algorithm

Second, based only on the observed abundances of the candidate pathogenic bacteria, we trained the Random Forest with balanced subsampling to generate an accurate classifier. Finally, we used this classifier to test the other one-third of normal and sepsis samples reserved for this purpose. The above pipeline was repeated 1000 times through bootstrap. As shown in Fig. 3a, the average out-of-bag error (OOB error) was 0.16 when there were a sufficiently large number of decision trees (> 100). The performance of the diagnosis strategy is satisfactory, with an average AUC of 0.926, sensitivity of 0.91 and specificity of 0.83. As an alternative, we also tried a logistic regression approach as a comparison (average AUC 0.77, sensitivity of 0.71 and specificity of 0.80) (Fig. 3b). The ranked list of the candidate bacterial species with respect to their importance in the Random Forest model is provided in Additional file 2: Table S2.

For the validation of an independent dataset, the 118 healthy and 38 sepsis samples respectively from study 1 (No. PRJEB13247) and study 2 (No. EGAS00001001754) were used as the training set, and samples from study 3 (No. PRJNA507824) was set as an independent validation. The AUC shows that the proposed method also performs well in the independent dataset (Fig. 3c).

### Bacterial co-occurrence networks based on cfDNA

**Fig. 4:**
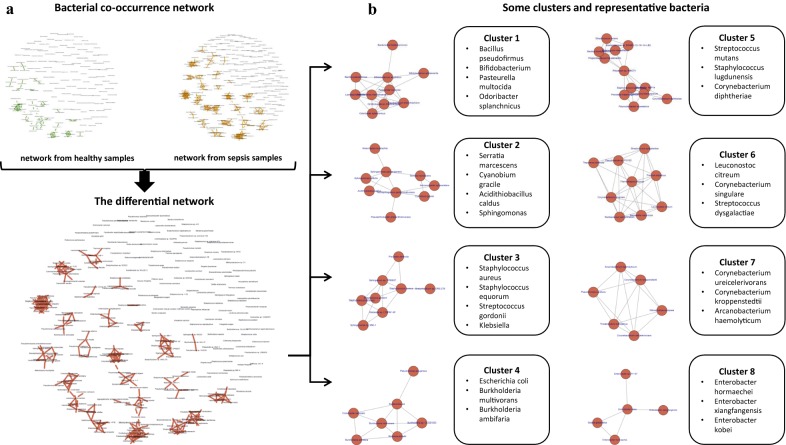
Bacteria co-occurrence networks constructed on the basis of cfDNA data from normal and sepsis samples. **a** The differential co-occurrence network describing associations between species that are only observed in the sepsis samples. **b** A partial list of clusters (connected components) from the differential network. For each cluster, the representative bacteria are listed

Using the bacterial abundance matrices from 78 healthy and 25 sepsis samples for training, we constructed two bacterial co-occurrence networks (Fig. 4a). Each network contains 224 nodes, representing the 224 candidate pathogenic bacteria that were selected for having significantly different abundance distributions between healthy and sepsis samples. As mentioned above, blood can contain cfDNA fragments released by the bacteria inhabiting all human body sites. Thus, we expect the co-occurrence networks of healthy and sepsis samples to include some associations among “harmless” species that are generally not involved in sepsis. In order to focus on sepsis-specific associations, we generated a differential network by excluding from the sepsis co-occurrence network all association patterns also found in the healthy co-occurrence network (Fig. 4a). We found 19 clusters (Fig. 4b) of species in the differential network, which are the strongly connected components visible in Fig. 4a. In the 25 sepsis samples, all the species in a cluster are strongly correlated in terms of their abundance levels. The detailed cluster information is provided in Additional file 3: Table S3.

In order to analyze the biological features of the clusters, we characterized the species in each one according to three aspects: respiration mode, metabolic habitat, and growth rate.

First, among all candidate pathogen species, 35.52%, 3.66%, and 52.12% are anaerobic, aerobic, and facultative respectively (the remaining 8.7% are unknown). Most of the clusters show similarity in terms of respiration mode: 9 clusters exhibit a preference for facultative species (clusters 3, 5, 6, 10, 14, 15, 16, 17 and 19), and 7 clusters exhibit a preference for anaerobic species (clusters 1, 2, 7, 11, 12, 13 and 18). The few anaerobic species in the sample do not dominate any cluster.

Second, before causing infection in blood, these bacteria usually originate in specialized metabolic environments. Bacterial metabolic habitats are divided into 4 types: host-associated, terrestrial, aquatic, and diverse. The species in clusters 3, 4, 5, 9, 14, 15, 17, 18, and 19 are mainly host-associated, the species in cluster 10 are mainly terrestrial, the species in cluster 3 are mainly aquatic, and clusters 1, 6, 7, 10, 12, 13, 16 contain species from diverse metabolic environments.

Third, bacterial growth is significantly correlated with metabolic variability and the level of co-habitation. Doubling-time data have led to the important finding that variations in the expression levels of genes involved in translation and transcription influence growth rate [34, 35]. We partition the clusters into two groups according to the doubling time of their member species: “fast” and “slow” growing clusters are those whose median duplication time is shorter or longer than the mean over all species by at least one standard deviation [36]. The median doubling time for species distributed in cluster 6, 7, 11 and 13, is larger than 1 (fast growing clusters), while doubling time for members in cluster 1, 3, 4, 5, 15, 16 is smaller than 0.6 (slow growing clusters). Note that fast growth rates are typical of species that exhibit ecological diversity, so the identification of “fast” clusters accords with the metabolic habitats analyzed in the previous paragraph.

For the pathogens of each cluster, a specific therapy of antibiotics could be provided [37]. A list of possible antibiotics that might be used for each of cluster is shown in Additional file 3: Table S3.

### Inferring missing bacteria from identified species

A given patient with sepsis can carry multiple pathogens [38]. Therefore, knowledge of all bacteria present is crucial if we are to provide fast and effective antibiotic treatment. At the same time, the pathogenic species span a wide range of growth strategies and environmental requirements (such as aerobic or anaerobic, acidity, etc.), which makes it difficult to detect all species in a single culture. Moreover, due to the limited volume of a blood sample, not all pathogenic species can be identified from cfDNA. In short, unobserved bacterial species are a major obstacle to effective treatment.

Based on the bacterial co-occurrence network, it is possible to infer missing bacterial species from the identified species. Specifically, having constructed a bacterial co-occurrence network, we know that some species usually have consistent abundance levels in sepsis samples. Thus, when some species from a cluster are identified in a sepsis sample, statistically it is highly probable that all members of the cluster are present. We can infer the presence of “missing” bacteria in this way, if the missing bacteria belong to a cluster.

**Fig. 5:**
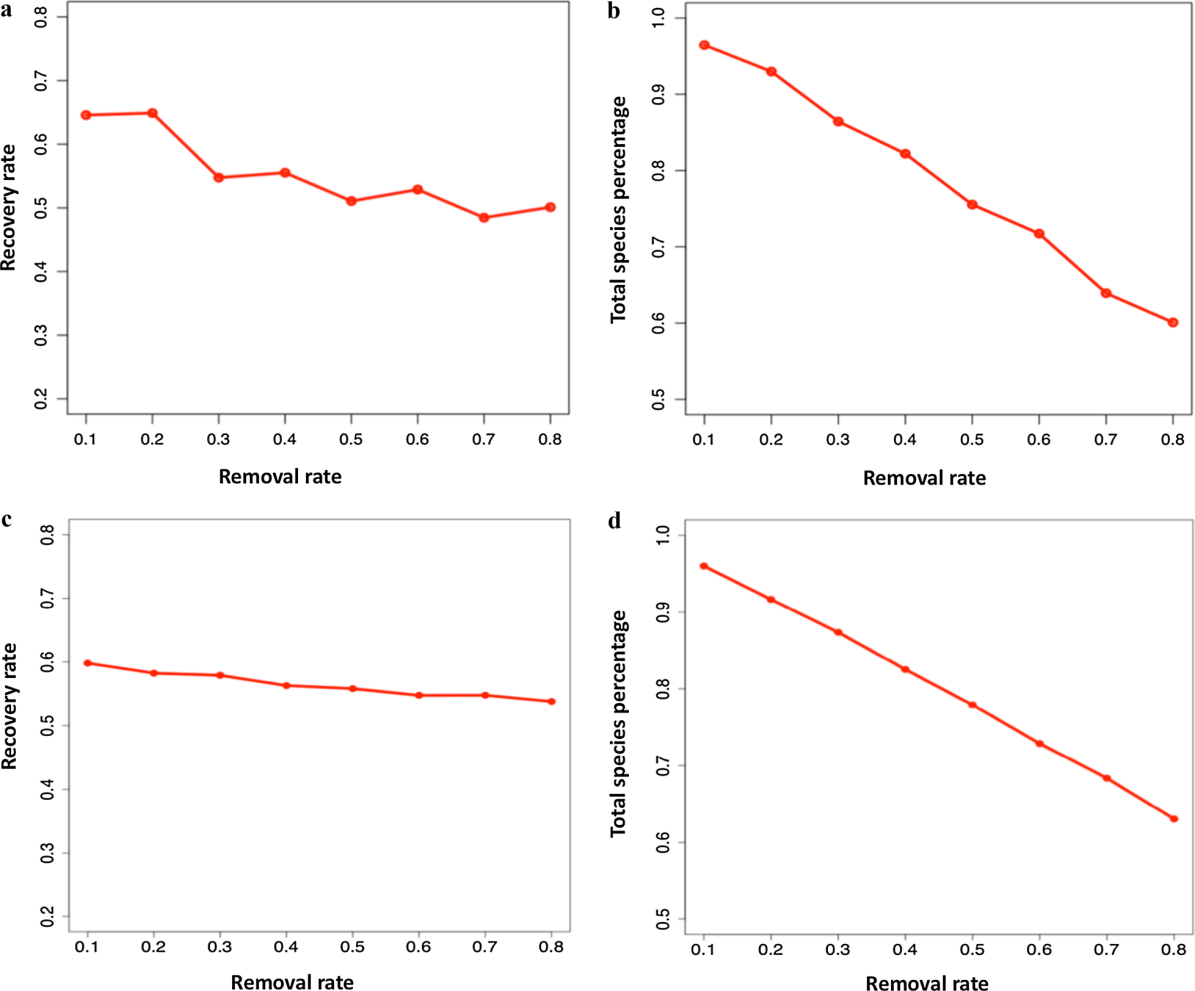
The performance of species inference based on the bacteria co-occurrence network. The curve shows the average recovery rate. For each testing sepsis sample, we performed 1000 trials. In each trial, we randomly removed 10–80% of observed bacterial species then inferred the presence of missing species from the co-occurrence network. The x-axis represents the removal percentage. **a** The y-axis represents the percentage of inferred species that were removed in the cross-validation. **b** The y-axis represents the total percentage of identified species for the cross-validation, including both inferred species and those that were never removed. **c** The y-axis represents the percentage of inferred species that were removed in for the validation based on an independent data. **d** The y-axis represents the total percentage of identified species for the validation of an independent data

To test the effectiveness and robustness of this bacteria-inferring scheme, a certain percentage of species were randomly removed from the identified species pool for each sample for both cross-validation and the validation of an independent dataset. We tried to infer the presence of the missing bacteria from the remaining species, based on the bacterial co-occurrence network. Figure 5a, c show that the recovery rate is about 50–60%, decreasing gradually with higher removal rates. And the overall results are quite satisfactory, as seen in Fig. 5b, d. The total number of species recovered (including those not randomly removed) is still 60%, even when 80% of the observed species were randomly removed. These results demonstrate the effectiveness of a bacterial co-occurrence network to infer the presence of unobserved bacteria from identified species. This method has great potential, especially in cfDNA-based analysis, because in a 10 ml blood sample there is a very limited amount of cfDNA, and only a small proportion of that is microbial cfDNA.

## Discussion

Sepsis or bacteremia is a common and serious disease, which requires a quick and accurate diagnosis and identification of pathogens in order to select the appropriate antibiotic treatment. The standard procedure includes confirmatory tests (e.g., recognizing clinical signs and symptoms, Procalcitonin test, SeptiCyte test) and culture-based pathogen identification. As reported by recent studies, the culture-based diagnosis is time-consuming and requires strict anaerobic conditions to promote bacterial growth. Moreover, only a third to a half of people with sepsis yield positive blood cultures [6]. In this work, we developed a noninvasive approach to sepsis diagnosis and pathogen identification using cfDNA sequencing data mapped to bacteria genomes. This approach does not require cultivation, greatly enhancing the efficiency of diagnosis. Our method achieves AUC of 93% (cross-validation) and 88% (the independent validation), which outperforms by far the blood culture approach. The comparison between the bacteria inferred by our method and those from blood culture are demonstrated in Additional file 4: Table S4. It is seen that the 84.69% pathogenic bacteria detected by by blood culture agree with those by our method.

The estimated turn-around time of our method is about a day, the time currently required for cfDNA sequencing. This time will be further reduced in the future, due to technology improvements and faster sequencing. Therefore, our method may provide accurate and rapid identification of sepsis samples.

Further, the differential bacterial co-occurrence network supports an inference scheme to find “missing” bacteria based on observe and identified species. This approach permits comprehensive profiling of all bacteria involved in the infection process. It is particularly applicable to the scenario where only small blood samples (e.g. 10 ml) are available, and many bacterial species go unobserved. This combination of rapid sepsis diagnosis and pathogen inference is especially suitable for cfDNA-based diagnosis, which is now accepted as a promising, noninvasive tool in disease detection.

## Conclusion

In this work, we identified sepsis-causing bacteria from limited sepsis samples. Additional sepsis-causing species can be identified and more accurate co-occurrence networks can be generated as more and more whole-genome deep sequencing data become available, from healthy and sepsis cohorts. Therefore, we expect this approach to achieve higher accuracy in the near future. In addition, we expect that a time series of blood samples taken from patients can further enhance the prognosis and diagnosis of sepsis. This research is merely a first step towards diagnosing sepsis using cfDNA, in that it demonstrates a new way to employ cfDNA sequencing data with a network approach to achieve rapid disease diagnosis.

## Supplementary information

**Additional file 1: Table S1.** All species with P-values.

**Additional file 2: Table S2.** Importance of candidate bacterial species by Random Forest.

**Additional file 3: Table S3.** The cluster information of the bacterial co-occurrence network.

**Additional file 4: Table S4.** Inferring bacteria of the bacterial co-occurrence network and blood culture.

## Data Availability

The raw sequencing reads were derived from two published data sources: European Nucleotide Archive (https://www.ebi.ac.uk/ena/data/view/PRJEB13247, https://www.ncbi.nlm.nih.gov/Traces/study/?acc=SRP172792) and European Genome-phenome Archive (https://www.ebi.ac.uk/ega/studies/EGAS00001001754).
